# A regulatory GhBPE-GhPRGL module maintains ray petal length in *Gerbera hybrida*

**DOI:** 10.1186/s43897-022-00030-3

**Published:** 2022-04-08

**Authors:** Rui Jiang, Weichao Yuan, Wei Yao, Xuefeng Jin, Xiaojing Wang, Yaqin Wang

**Affiliations:** 1 Guangdong Provincial Key Laboratory of Biotechnology for Plant Development, School of Life Sciences, South China Normal University, Guangzhou, 510631 China; 2 Guangdong Laboratory for Lingnan Modern Agricultural, Guangzhou, 510642 China

**Keywords:** GhBPE-GhPRGL, GA, JA, ray petal elongation, *Gerbera hybrida*

## Abstract

The molecular mechanism regulating petal length in flowers is not well understood. Here we used transient transformation assays to confirm that *GhPRGL* (proline-rich and GASA-like)—a GASA (gibberellic acid [GA] stimulated in Arabidopsis) family gene—promotes the elongation of ray petals in gerbera (*Gerbera hybrida*). Yeast one-hybrid screening assay identified a bHLH transcription factor of the jasmonic acid (JA) signaling pathway, here named GhBPE (BIGPETAL), which binds to the *GhPRGL* promoter and represses its expression, resulting in a phenotype of shortened ray petal length when *GhBPE* is overexpressed. Further, the joint response to JA and GA of *GhBPE* and *GhPRGL*, together with their complementary expression profiles in the early stage of petal growth, suggests a novel GhBPE-GhPRGL module that controls the size of ray petals. GhPRGL promotes ray petal elongation in its early stage especially, while GhBPE inhibits ray petal elongation particularly in the late stage by inhibiting the expression of *GhPRGL*. JA and GA operate in concert to regulate the expression of *GhBPE* and *GhPRGL* genes, providing a regulatory mechanism by which ray petals could grow to a fixed length in gerbera species.

**Supplementary Information:**

The online version contains supplementary material available at 10.1186/s43897-022-00030-3.

## Core

GhBPE maintains a fixed ray petal length in gerbera by repressing *GhPRGL* transcription during petal growth and development. GhPRGL and GhBPE are subject to common dynamic regulation by the plant hormones GA and JA, and their opposing functions ultimately determine petal length. Consequently, we propose a novel GhBPE-GhPRGL module that regulates the size of ray petals.

## Gene & accession numbers

Sequence data from this article can be found in the Arabidopsis Genome Initiative or GenBank/EMBL databases under the following accession numbers: AtBPEp, NP_849829.1; AtBPEub, NP_564749.1; GhBPE, GACN01010423.1; LsbHLH094-like, XP_023740329.1; HabHLH094, XP_021973982.1; TobHLH, PON53727.1; PabHLH, PON67443.1; MeBHLH089-like, XP_021616639.1; and CcBHLH79, XP_020238985.1.

## Introduction

Flowers are the sexual reproductive organs of angiosperms and are composed of petals of various shapes, sizes, and colors. As one of the most important components of flower organs, petals play an indispensable role in protecting the stamen and pistil, attracting insect pollinators, and facilitating the sexual reproduction of plants (Irish, 2010; Hermann and Kuhlemeier, 2011). When a plant shifts from vegetative to reproductive growth, flower buds develop instead of vegetative buds (Vaughan, 1955). The plant’s apical meristem transforms into the inflorescence meristem; at the latter’s edge forms the flower meristem, which in turn forms the floral primordium. Under the control of organ identity genes, the floral primordium sequentially produces sepals, petals, stamens, and pistils, which together form the flower (Smyth et al., 1990; Coen and Meyerowitz, 1991; Krizek and Fletcher, 2005). Early flower development refers to the chain of events spanning from flower bud’s initiation to its opening, which may be divided into 12 stages. The first of these is the initiation of a floral buttress on the flank of the apical meristem, followed by other key stages that include the formation of the floral primordia, the formation of different types of floral organs, and finally the opening of the flower bud (Smyth et al., 1990). Bud opening marks the beginning of late flower development, where petals begin to elongate until they are fully open and reach a fixed size (Müller, 1961). In gerbera (*Gerbera hybrida*), according to the characteristics of ray petal length and anthocyanin pigment accumulation, late flower development can be divided into six stages (S1–S6) (Meng and Wang, 2004).

Petal growth and development depends on cell division and proliferation and cell expansion resulting from the expression and coordinated interaction of a series of genes (Irish, 2008; Powell and Lenhard, 2012). In Arabidopsis, certain known genes, such as *RABBIT EARS* (*RBE*), *AINTEGUMENTA* (*ANT*), *AUXIN REGULATED GENE INVOLVED IN ORGAN GROWTH* (*AGROS*), and *JAGGED* (*JAG*), play a role early on in flower development, mainly by promoting cell division and proliferation, whereas other genes like *ARF8* (AUXIN RESPONSE FACTOR8), *BPEp* (*BIGPETALp*), and *MED25* (Mediator complex subunit 25) inhibit cell expansion in later stages (Mizukami and Fischer, 2000; Hu et al., 2003; Dinneny et al., 2004; Weiss et al., 2005; Breuninger and Lenhard, 2010; Xu and Li, 2011). In ornamental plants, the overexpression of *RhNAC100* (no apical meristem [NAM], Arabidopsis transcription activation factor [ATAF], and cup-shaped cotyledon [CUC]) significantly reduces petal size by inhibiting cell expansion during the late stages of rose petal growth and development (Pei et al., 2013). Overexpression of *CmTCP20* (TEOSINTE BRANCHED1/CYCLOIDEA/PROLIFERATING CELL FACTORs) can promote petal elongation by suppressing cell division and promoting the cell expansion of chrysanthemum petals (Wang et al., 2019a, b). Heterologous transformation of *ZmGS5* from maize, a cereal crop, increases petal size in Arabidopsis by promoting cell expansion (Wang et al., 2021). In addition to these genes that regulate cell size, petal growth from floral organ primordia is also affected by a plant’s external conditions and internal hormone levels, which influence its normal growth and development (Weiss et al., 2005). In recent years, mounting studies have found that plant hormones participate in the gene regulation network governing petal shape (Weiss et al., 2005; Han et al., 2017; Wang et al., 2017; Huang et al., 2017, 2020). However, until now, the molecular mechanisms that determine petal size have remained mostly unknown, although a number of genes have been identified and their regulatory mechanisms studied in some detail.

Phytohormones can positively or negatively regulate the length of the petal to maintain its fixed size in different plant species. For example, auxin promotes petal elongation in the chrysanthemum ‘Jinba’ (Wang et al., 2017); cytokinins regulate the size of the flower organ by promoting the proliferation of petal cells, resulting in larger petals in Arabidopsis (Bartrina et al., 2011); and, in gerbera, brassinolide (a common active ingredient of BRs [brassinosteroids]) promotes cell elongation in different regions of the petal (Huang et al., 2017). Conversely, ethylene inhibits cell expansion in both gerbera and rose petals (Ma et al., 2008; Huang et al., 2020), while abscisic acid (ABA) inhibits cell elongation and has an antagonistic effect on gibberellic acid (GA) in gerbera (Li et al., 2015). Another plant hormone that is indispensable for plant growth and development is jasmonic acid (JA), a lipid-derived phytohormone involved in the regulation of a variety of biological processes (Liu and Timko, 2021), such as root growth (Staswick, Su and Howell, 1992; Gutierrez *et al.*, 2012), leaf senescence (Xiao et al., 2004; Jiang et al., 2014), plant defense (Wang et al., 2019a, b; Ali and Baek, 2020), and secondary metabolite biosynthesis (Franceschi and Grimes, 1991; De Geyter et al., 2012). In particular, JA also regulates various aspects of flower development (Ishiguro et al., 2001; Ito et al., 2007; Oh et al., 2013; Yu et al., 2018; Acosta and Przybyl, 2019; Guan et al., 2021). Although JA’s synthesis and signaling are crucial for all aspects of the plant life cycle, its role in regulating petal size has not been extensively studied. However, it is known that JA stimulates the expression of the bHLH transcription factor (TF) BPEp, which acts downstream of *OPR3* in the JA signaling pathway, thereby inhibiting cell expansion and resulting in a smaller petal phenotype in Arabidopsis (Szécsi et al., 2006; Brioudes et al., 2009).

Members of the GASA (gibberellic acid stimulated in Arabidopsis) family are proline-rich cell wall proteins featuring a highly conserved structure, including 12 cysteine residues at the C-terminal end, which are involved in the process of cell extension and cell division, and these proteins play pivotal roles in root formation and elongation, stem growth, petal growth, seed germination, and fruit ripening (Ben-Nissan et al., 2004; Roxrud et al., 2007; Zhang et al., 2009; Rubinovich and Weiss, 2010). *Gerbera hybrida*, which like all gerbera belongs to the Asteraceae family, is among most prized cut flowers worldwide, and both petal size and color are crucial to its commercial and ornamental value (Bhatia et al., 2009; Mosqueda Frómeta et al., 2017). Currently, two GASA family genes in gerbera, named *GEG* (*Gerbera* homolog of *GAST1* gene) and *GhPRGL* (proline-rich and GASA-like), have been cloned and their expression patterns studied (Kotilainen et al., 1999; Peng et al., 2008, 2010). Interestingly, these two genes show completely opposite expression profiles during stage 1 to stage 6 (S1–S6) of petal growth. Our prior research demonstrated that both GhMIF (Mini Zinc-Finger protein) and GhEIL1 (Ethylene Insensitive 3-like 1) are capable of binding to the *GEG* promoter (Han et al., 2017; Huang et al., 2020), thus ensuring that *GEG* is highly expressed in the late stages of petal growth, when it inhibits ray petal elongation (Kotilainen et al., 1999). The *GhPRGL* gene, which is closely related to *AtPRGL* of Arabidopsis, is highly expressed during S1–S3, but its expression gradually declines with continued petal growth (Peng et al., 2010). Hence, one hypothesis worth investigating, along with the regulatory mechanism involved, is that *GhPRGL* has an opposing function to *GEG* in the growth and development of petals.

Here, we report evidence confirming that *GhPRGL*, a novel petal size regulation gene, can promote the elongation of ray petals in gerbera. To explore the underlying molecular mechanism, we identified a bHLH TF of the JA signaling pathway, here named GhBPE, which can bind to the *GhPRGL* promoter. Further experiments showed that GhBPE suppressed *GhPRGL* expression to reduce ray petal length. Based on our results, we identified a GhBPE-GhPRGL module that is crucial for the regulation of ray petal length in gerbera: *GhPRGL* promotes ray petal elongation, especially in S1–S3, whereas GhBPE inhibits ray petal elongation, especially in S3–S6, by inhibiting the expression of *GhPRGL*; JA and GA work together to regulate the expression of the GhBPE-GhPRGL module. Thus, as part of different phytohormone signaling pathways, GhBPE and GhPRGL jointly govern the growth and development of ray petals such that their fixed length is maintained in gerbera flower.

## Results

### GhPRGL, a GASA protein, promotes ray petal elongation in gerbera

**Fig. 1:**
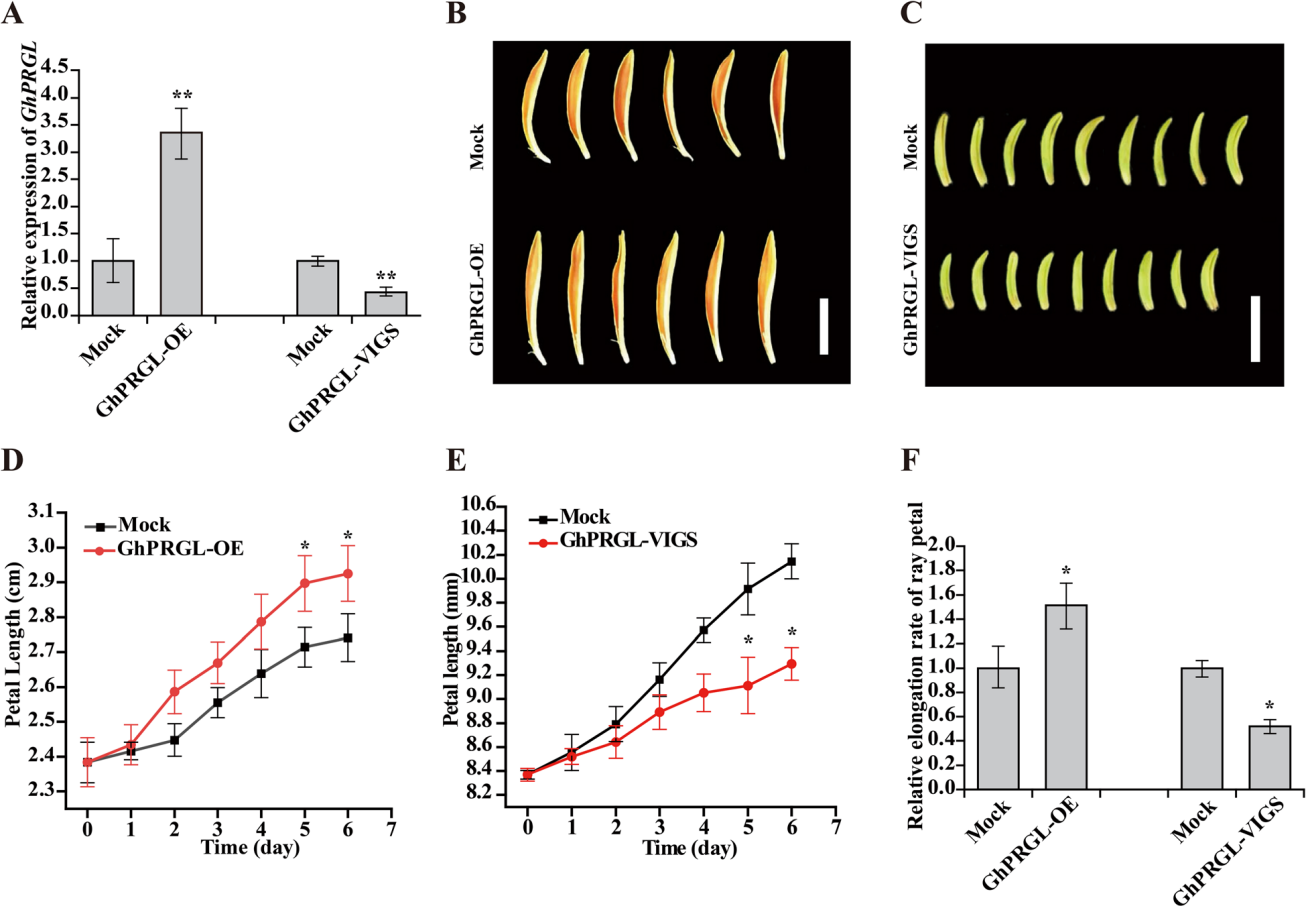
*GhPRGL* promotes ray petal elongation of gerbera. **A** Expression levels of *GhPRGL* in the mock and *GhPRGL*-OE, the mock and *GhPRGL*-VIGS are shown from left to right, respectively. Ray petal phenotypes of (**B**) *GhPRGL*-OE and (**C**) *GhPRGL*-VIGS after 6 days of culturing. Time-course dynamics of ray petal length in (**D**) *GhPRGL*-OE and (**E**) *GhPRGL*-VIGS after transformation (*n* = 20). **F** Relative elongation rate of ray petals in each treatment. Scale bars are 1 cm in (**B**) and (**C**). Each observation was performed using at least three biological replicates Tukey’s HSD: * P < 0.05, ** P < 0.01

Our earlier research showed that *GhPRGL*, a GASA protein, is highly expressed during S1–S3 (Peng et al., 2010). To investigate the function of *GhPRGL*, transient transformation assays were performed with S3 ray petals for *GhPRGL*-overexpression (*GhPRGL*-OE) and with S1.5 ray petals for *GhPRGL*-silencing (*GhPRGL*-VIGS) for use in the experiments. We used petals from S3 because the transition between cell proliferation and cell expansion occurs at this stage; hence, S3 petals are suitable for studying both petal growth and petal size (Laitinen et al., 2007; Li et al., 2015). We selected S1.5 ray petals for the *GhPRGL*-silencing experiments because *GhPRGL* expression is very low in S3, rendering its results less clear. The present results showed the expression level of *GhPRGL* significantly increased (by ~3.36-fold) in *GhPRGL*-OE petals and decreased (by ~0.42-fold) in *GhPRGL*-VIGS petals when compared with their counterpart mock controls, indicating the successful overexpression and silencing of *GhPRGL*, respectively (Fig. 1A; Fig. S1). Further, ray petal length was promoted in *GhPRGL*-OE petals and inhibited in *GhPRGL*-VIGS petals (Fig. 1B, C). By measuring ray petal length, *GhPRGL*-OE petals were significantly elongated compared with mock, while petal length was inhibited by silencing *GhPRGL* (Fig. 1D, E). In addition, ray petal elongation rates were significantly higher in *GhPRGL*-OE petals and lower in *GhPRGL*-VIGS petals relative to their respective mock control (Fig. 1F). Considering altogether the statistical results of transgenic petal cell length, cell width, and cell numbers per unit area (Fig. S2), we posited that *GhPRGL* promotes ray petal elongation by regulating cell elongation.

### Analysis of the *GhPRGL* promoter

**Fig. 2:**
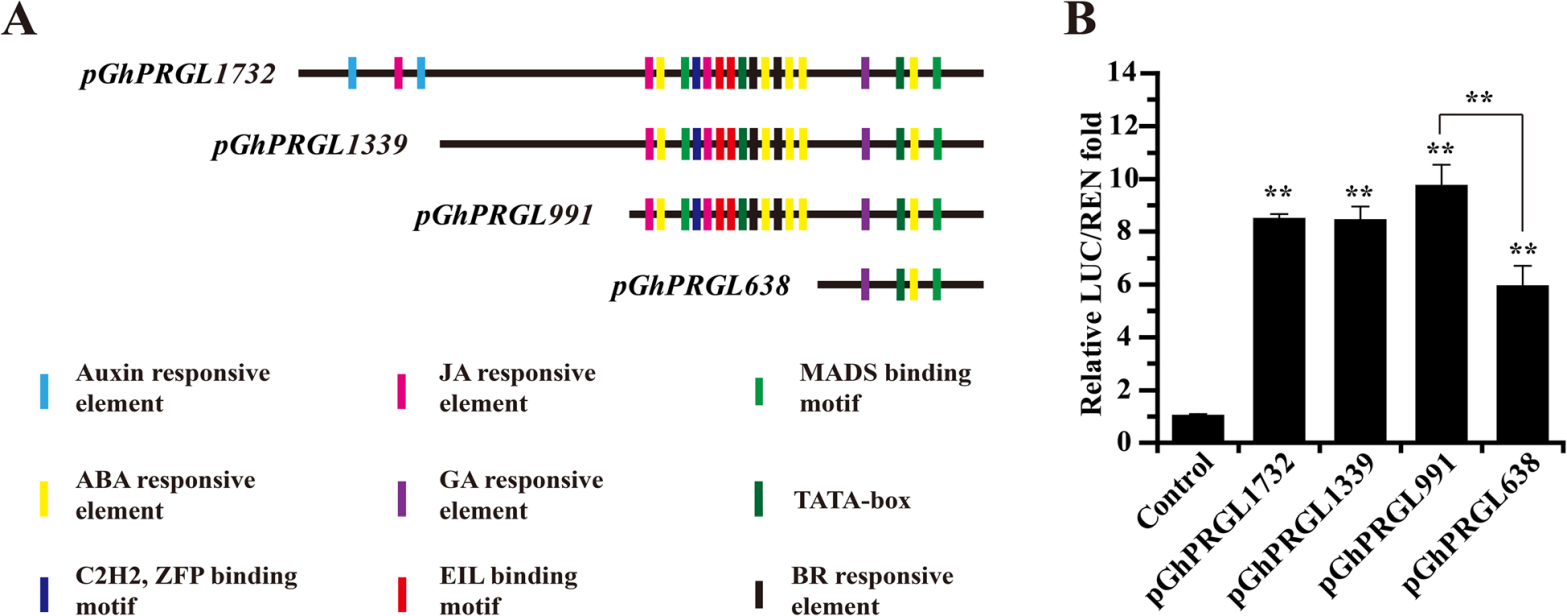
Prediction of TF binding sites in the promoter of *GhPRGL* and experimental verification of the core region that affects gene expression. **A** Four serial 5’-deletion fragments (pGhPRGL1732, pGhPRGL1339, pGhPRGL991, and pGhPRGL638) were generated, with different colors denoting the different binding elements. On the left side of each fragment is its length. **B** Relative reporter activity of each promoter fragment in the dual luciferase assay. The control LUC/REN fold value was set to 1.0. Values are the mean ± SD of three biological replicates. Tukey’s HSD: ** P < 0.01

To understand how *GhPRGL* is regulated, its full-length promoter—positioned 1732 bp upstream of the first nucleotide (designated as +1) of the cDNA ORF—was amplified using Hi-TAIL PCR (Table S1). Both the PLACE and PlantCARE databases of *cis*-acting regulatory elements were searched for motifs in the *GhPRGL* promoter sequence. In addition to conserved promoter elements such as the TATA-box and CAAT-box, the promoter region contained hormone (GA, JA, ABA, BR, and auxin)-responsive elements and floral organ development-related elements, including those known to bind the MADS, EIL, and ZFP TFs (Fig. 2A). In Arabidopsis, each *GASA* gene has 1–5 ABRE (ABA-responsive element) and 1-4 GARE (GA-responsive element) *cis*-elements in its promoter sequence, as well as other types of ABA- and GA-related *cis*-elements (Zhang and Wang, 2008). Not only did we find that *GhPRGL* had ABA- and GA-responsive elements in its promoter, consistent with the findings from Arabidopsis, but the gerbera gene also harbored JA-responsive elements, implying that *GhPRGL* activity may be co-regulated by multiple hormones.

To identify which regions of the promoter controlled *GhPRGL*’s expression level, serial 5’-deletion fragments (*pGhPRGL1732*, *pGhPRGL1339*, *pGhPRGL991*, and *pGhPRGL638*) were generated and tested using a dual-luciferase assay in gerbera protoplasts. All four constructs showed significantly higher LUC/REN ratios (5.89–9.71-fold) than the controls, suggesting these promoter regions all contain elements important for *GhPRGL* expression. The construct *pGhPRGL991* (9.71-fold > the control) was distinguished by markedly greater reporter activity than *pGhPRGL638* (5.89-fold) (Fig. 2B); this indicated the region of the *GhPRGL* promoter from –991 to –638 bp (*pGhPRGL353*) is crucial for *GhPRGL* expression.

### GhBPE, a bHLH TF, binds to the promoter of *GhPRGL* and represses its expression

**Fig. 3:**
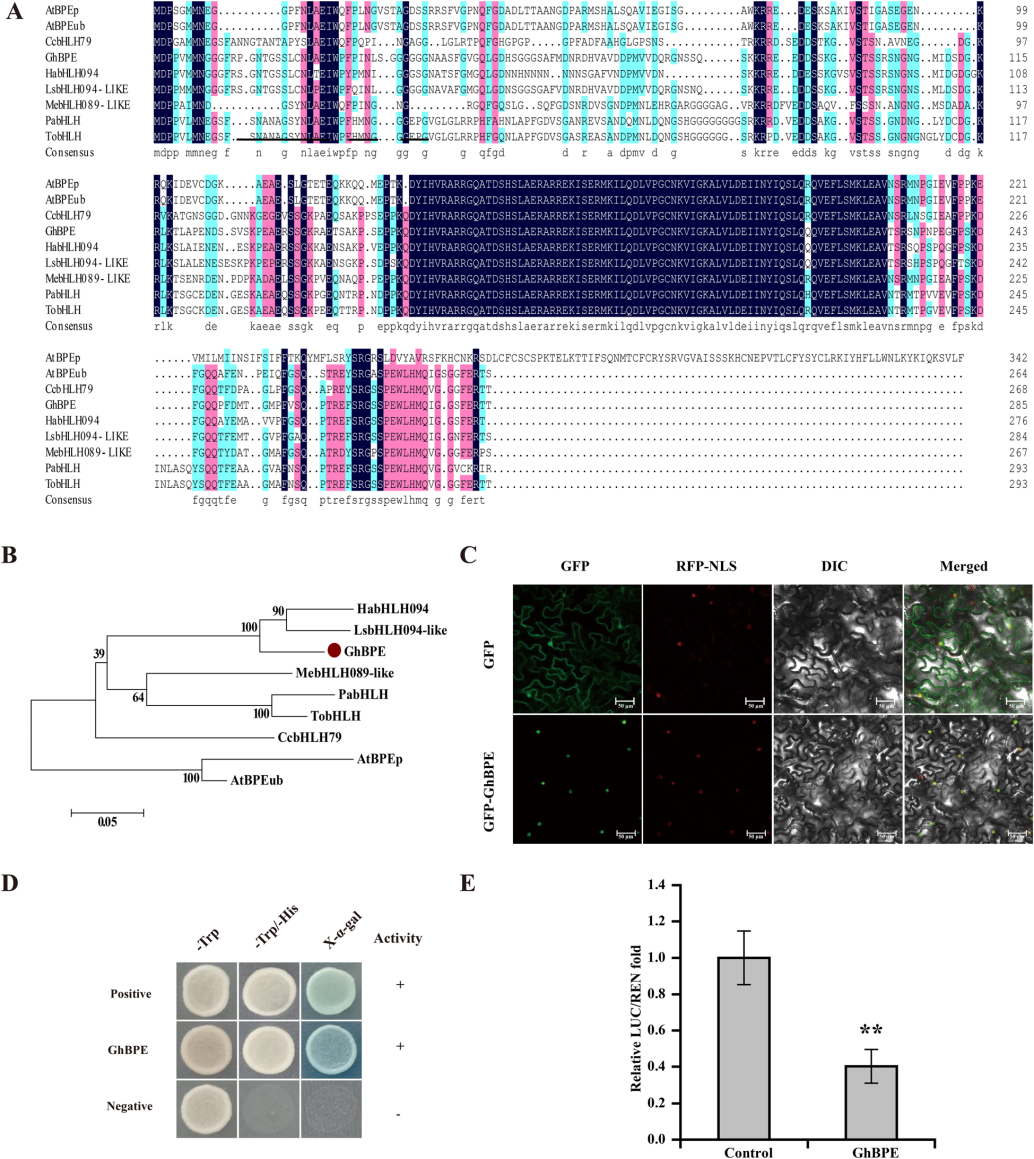
Biological function analysis of GhBPE. **A** Amino acid alignment of BPE proteins in various plant species. The black line above the sequence indicates the bHLH domain. **B** Phylogeny of the bHLH family genes in different species. The bootstrap values shown indicate the robustness of each branch. The scale bar corresponds to 0.05 substitutions per site. Amino acid sequences were used for the amino acid alignment and phylogeny analysis. AtBPEp (NP_849829.1) *Arabidopsis thaliana*; AtBPEub (NP_564749.1) *Arabidopsis thaliana*; LsbHLH094-like (XP_023740329.1) *Lactuca sativa*; HabHLH094 (XP_021973982.1) *Helianthus annuus*; TobHLH (PON53727.1) *Trema orientale*; PabHLH (PON67443.1) *Parasponia andersonii*; MeBHLH089-like (XP_021616639.1) *Manihot esculenta*; and CcBHLH79 (XP_020238985.1) *Cajanus cajan*. **C** Subcellular localization of GhBPE in tobacco leaves. GhBPE fused with GFP or GFP was transformed into tobacco leaves. Nuclei are represented by RFP-NLS. Merged images show the co-localization of NLS and GFP signals. Scale bar = 50 μm. **D** Transcription activation activity analysis of GhBPE. The vector pGADT7-largeT/pGBKT7-p53 and pGADT7-largeT/pGBKT7-laminC were transformed into yeast cells and used as negative and positive controls, respectively. The yeast strains were grown on SD/-Leu/-Trp, SD/-Leu/-Trp/-His, and SD/-Leu/-Trp/-His+X-α-gal solid media. **E** The transcriptional activity of GhBPE was analyzed using a dual-luciferase assay. The empty pBS vector, reporter and internal control vector were co-transformed as a control. The experimental results are conveyed using the mean ± SD of three biological replicates. Tukey’s HSD: * P < 0.05, ** P < 0.01

A bHLH TF (GACN01010423.1, GhBPE) was identified via yeast one-hybrid assay (Y1H) screening (see below); it contained an 858-bp ORF and encoded a protein of 286 amino acids (Table S1). GhBPE was first characterized by comparing its conserved amino acid sequences with bHLH family members of different species. The results showed that GhBPE, similarly to AtBPEp and AtBPEub, has a highly conserved bHLH domain at its N-terminus (Fig. 3A). A phylogenetic tree of GhBPE and other bHLH-like proteins revealed that GhBPE clustered with bHLH-like proteins from other Asteraceae species, such as *Helianthus annuus* and *Lactuca sativa* (Fig. 3B). Accordingly, the function of GhBPE may be similar to that of bHLH-like proteins from other Asteraceae species.

To examine the subcellular localization of GhBPE, GV3101 *Agrobacterium* cells harboring a GFP-GhBPE expression vector were introduced into *Nicotiana benthamiana* leaves containing the nucleus marker RFP-NLS. This experiment indicated that GhBPE was located in the nucleus (Fig. 3C). A yeast two-hybrid (Y2H) experiment showed that GhBPE has a self-activating activity, because a blue color was detected when X-α-gal was added to the synthetic dropout medium (Fig. 3D). Next, a dual-luciferase assay was used to verify the TF activity of GhBPE (Fig. S3). The luciferase activity of the GhBPE construct was significantly lower (~0.4-fold) than that of the control (Fig. 3E), which suggests GhBPE is a TF that acts as a repressor.

**Fig. 4:**
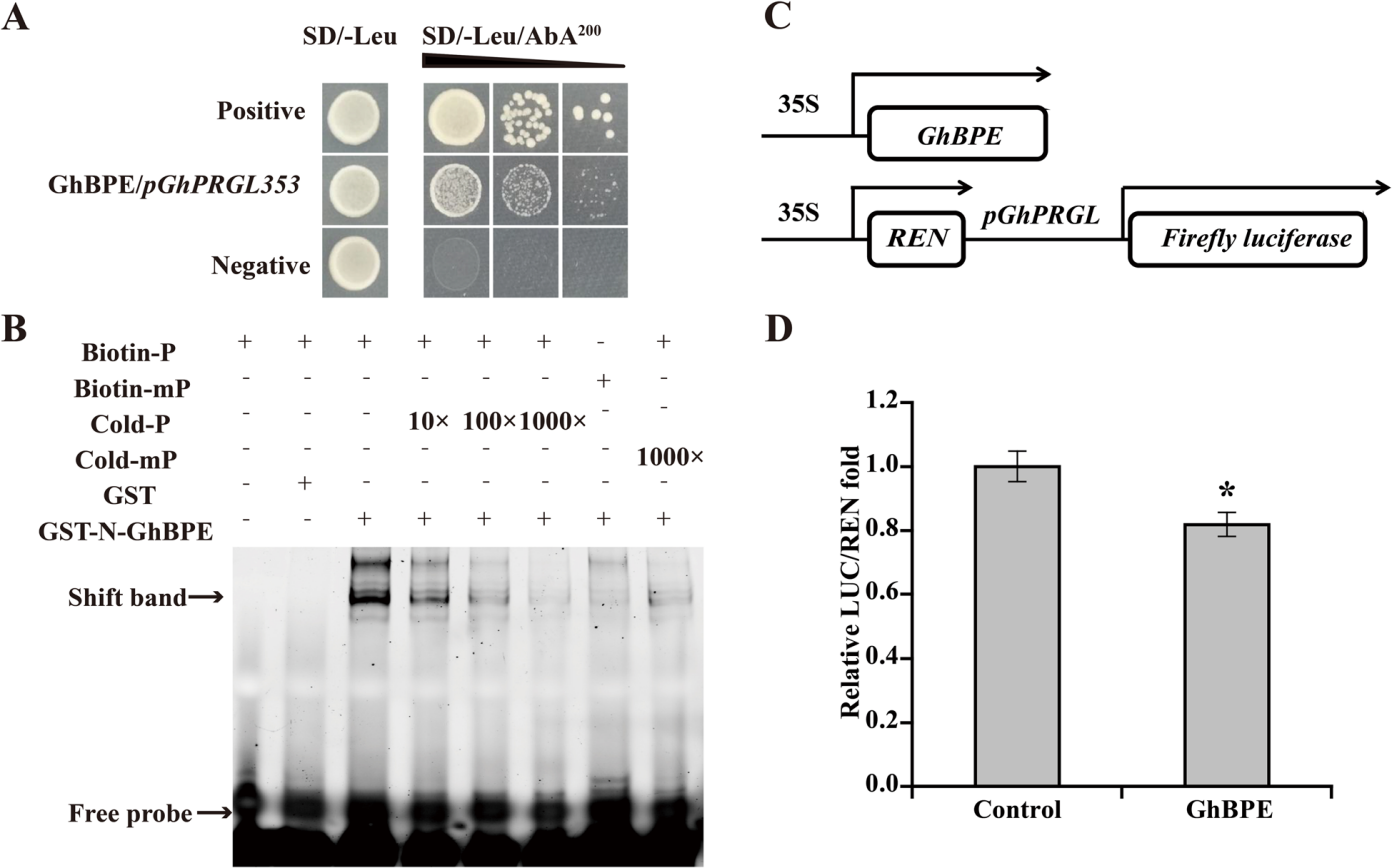
GhBPE binds to the *GhPRGL* promoter. **A** GhBPE binds to *GhPRGL* promoter according to the yeast one-hybrid results. The strain containing p53-AbAi/pGADT7-Rec-53 and pGhPRGL353-AbAi/pGADT7 vector served as the positive and negative controls, respectively. Black triangles indicate the fold-dilution of the bacterial concentration: 10^0^, 10^-1^, and 10^-2^, from the left to right. **B** GhBPE binds to the *GhPRGL* promoter by electrophoretic mobility shift assay (EMSA). Binding of GhBPE and biotin-labeled *GhPRGL* probes is indicated by black arrows. The "+" and "–" symbols represent the presence and absence of the corresponding components, respectively. **C** Schematic diagram of the dual-luciferase assay vectors for GhBPE binding with *GhPRGL* promoter. **D** GhBPE binds to the *GhPRGL* promoter by dual-luciferase assay. Empty effector vectors were the controls. The LUC/REN fold-change value of the control was set to 1.0. Values are the mean ± SD of three biological replicates. Tukey’s HSD: * P < 0.05

In the *GhPRGL* promoter’s region spanning –991 to –638 bp (*pGhPRGL353*), there are two JA responsive elements which are putative GhBPE binding *cis*-elements. The Y1H assay showed that yeast co-transformed with pGADT7-GhBPE and pGhPRGL353-AbAi was able to grow on SD-deficient medium, demonstrating specific binding of GhBPE to the *GhPRGL* promoter (Fig. 4A). An EMSA analysis was also carried out to confirm binding between GhBPE and the *GhPRGL* promoter. The EMSA showed the GST-N-GhBPE protein bound to the labeled –991 ~ –638 nt probe (including two JA-responsive elements) (Fig. 4B, lane 3), yet binding was competitively suppressed by 10-fold or higher for the unlabeled probe (Fig. 4B, lane 4–6), whereas GST-N-GhBPE protein could not bind to the mutant probe at all (Fig. 4B, lane 7–8); these results evinced that binding is specific. A dual-luciferase assay was then used to test whether GhBPE can affect the *GhPRGL* promoter (Fig. 4C). Co-transformation of *pGhPRGL1732* and GhBPE resulted in a lower LUC/REN ratio than the control, demonstrating that GhBPE binds to the *GhPRGL* promoter and inhibits the expression of *GhPRGL* (Fig. 4D). Overall, these results suggested that GhBPE is able to bind to the promoter of *GhPRGL* and transcriptionally repress its expression.

### GhBPE inhibits ray petal elongation by repressing the expression of *GhPRGL*

**Fig. 5:**
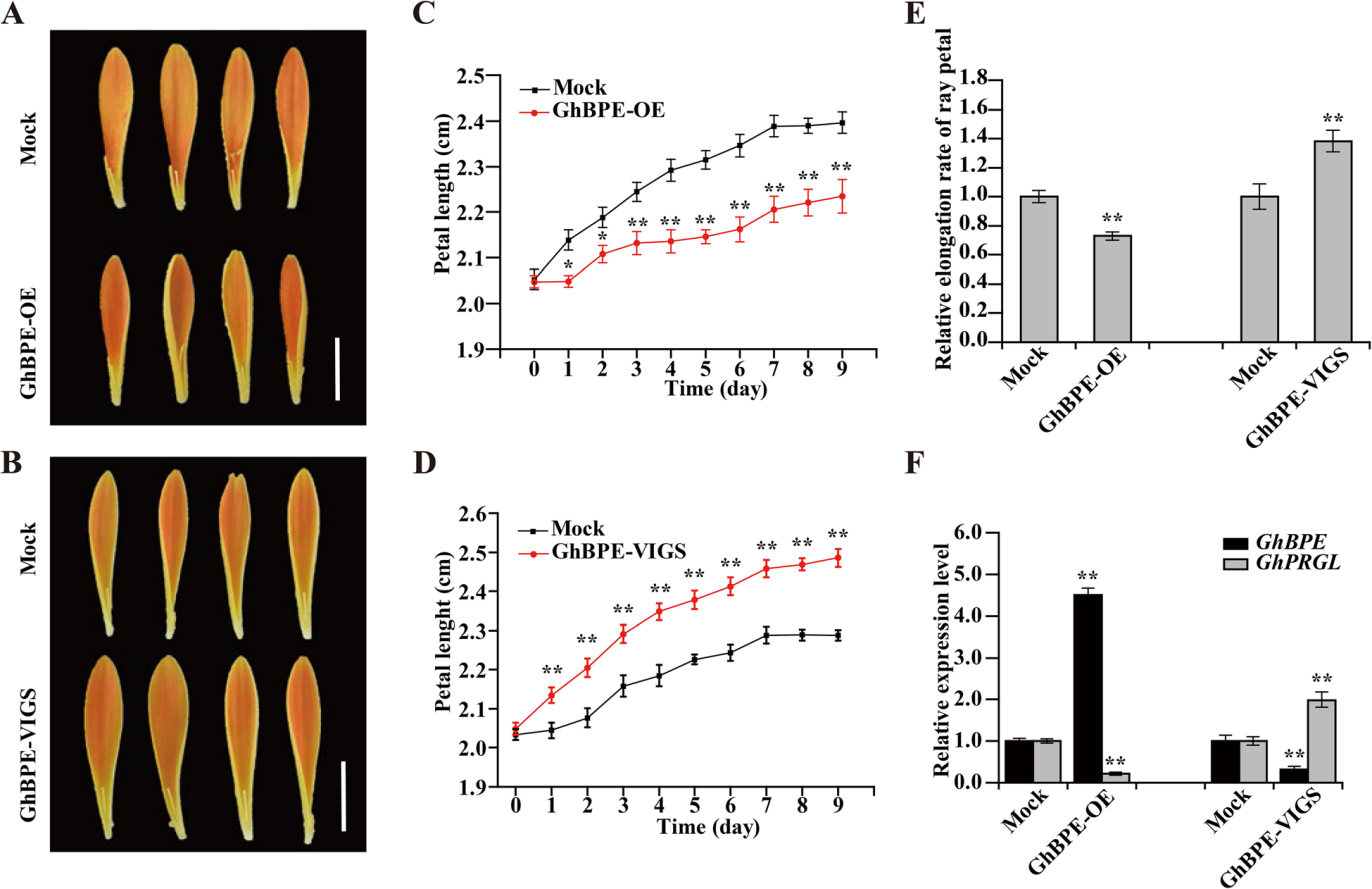
GhBPE inhibits ray petal elongation of gerbera. Ray petal phenotypes of (**A**) *GhBPE*-OE and (**B**) *GhBPE*-VIGS after 9 days of culturing. Time-course dynamics of ray petal length in (**C**) *GhBPE*-OE and (**D**) *GhBPE*-VIGS after transformation (*n* = 20). **E** The relative elongation rate of ray petals in each treatment. **F** Expression levels of *GhBPE* and *GhPRGL* in *GhBPE*-OE, *GhBPE*-VIGS, and the mock, respectively. Scale bars are 1 cm in (**A**) and (**B**). Each observation was performed with at least three biological replicates. Tukey’s HSD: * P < 0.05, ** P < 0.01

In Arabidopsis, AtBPEp inhibits the expansion of petals in the late phase of their growth (Szécsi et al., 2006). However, whether GhBPE plays a similar role in gerbera is unknown. To investigate this, we used transient overexpression and VIGS of *GhBPE* in ray petals. We found that ray petal elongation was significantly inhibited in *GhBPE*-OE petals, but was promoted in *GhBPE*-VIGS petals after 9 days of incubation (Fig. 5A–D). Accordingly, the relative petal elongation rate was 0.72-fold with overexpression, but reached 1.38-fold with gene silencing, vis-à-vis the mock controls (Fig. 5E; Fig. S4). We also used qRT-PCR to test the expression levels of *GhBPE* and *GhPRGL* in transiently transformed petals: *GhBPE* expression significantly increased (4.50-fold) in the OE samples but decreased (0.32-fold) in the silenced samples, when compared to the mock controls. By contrast, this trend was reversed for the expression of *GhPRGL*, it being substantially reduced in *GhBPE*-OE petals (~0.21-fold) and a markedly augmented in *GhBPE*-VIGS petals (~1.97-fold) (Fig. 5F).

**Fig. 6:**
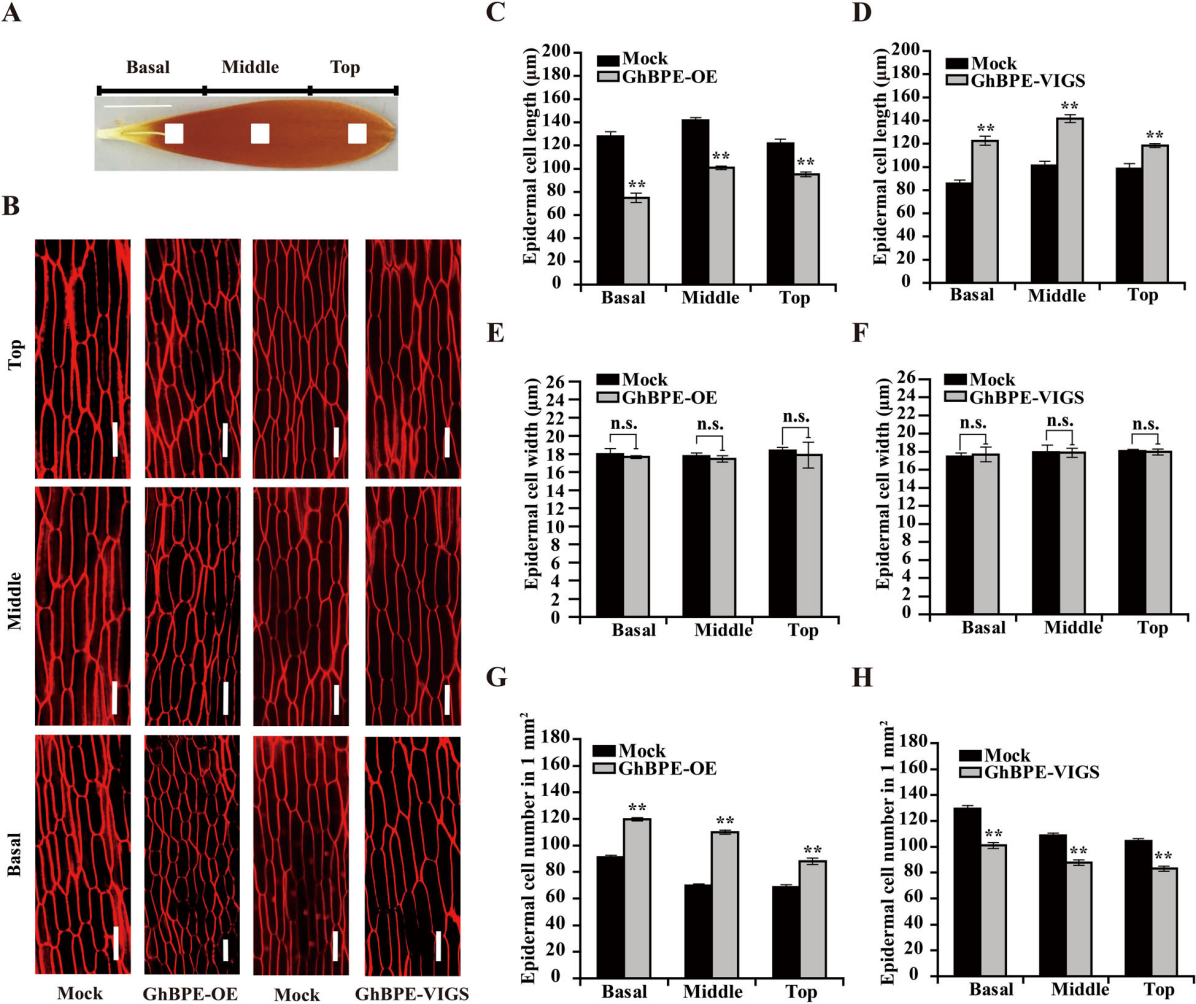
GhBPE inhibits the expansion of ray petal cells. **A** Blocks (each 1 mm^2^) at the center of the basal, middle, and top regions of ray petals were sampled for the morphological characterization of petal cells. **B** Images of the top, middle, and basal epidermal cells of the ray petals in the mock, *GhBPE*-OE, and *GhBPE*-VIGS after 9 days of culturing. Epidermal cell length of *GhBPE*-OE (**C**) and *GhBPE*-VIGS (**D**) ray petals in the top, middle, and basal region. Epidermal cell width of *GhBPE*-OE (**E**) and *GhBPE*-VIGS (**F**) ray petals in the top, middle, and basal region. Cell number of epidermal cells per unit area (1 mm^2^) of *GhBPE*-OE (**G**) and *GhBPE*-VIGS (H) ray petals in the top, middle, and basal region. Values are the mean ± SD of three biological replicates. Tukey’s HSD: ** *P* < 0.01. Scale bars are 1 cm (**A**) or 50 μm (**B**)

The final size of petals is determined by early stage cell division and late stage cell elongation (Krizek and Anderson, 2013). As Fig. S5 shows, the *GhBPE*-OE Arabidopsis lines underwent significant inhibition of petal extension and cell size. Accordingly, we measured the length, width, and number of epidermal cells in the top, middle, and basal regions of gerbera ray petals after 9 days of culturing (Fig. 6A, B). The epidermal cell length of *GhBPE*-OE petals was significantly shorter than the mock in all three regions, particularly in the basal sections (Fig. 6C). Also, the number of epidermal cells per unit area was much higher in *GhBPE*-OE (Fig. 6G). By contrast, the epidermal cell length of *GhBPE*-VIGS petals was significantly longer in all three regions (Fig. 6D), while the number of epidermal cells per unit area was reduced (Fig. 6H); however, the epidermal cell width of either transgenic petals were similar to that of the mock (Fig. 6E, F). These results suggested that GhBPE inhibited ray petal growth by regulating cell elongation. In general, we find that GhBPE inhibits ray petal elongation by suppressing the expression of *GhPRGL*.

### Regulation of petal elongation by the GhBPE-GhPRGL module is jointly mediated by JA and GA

**Fig. 7:**
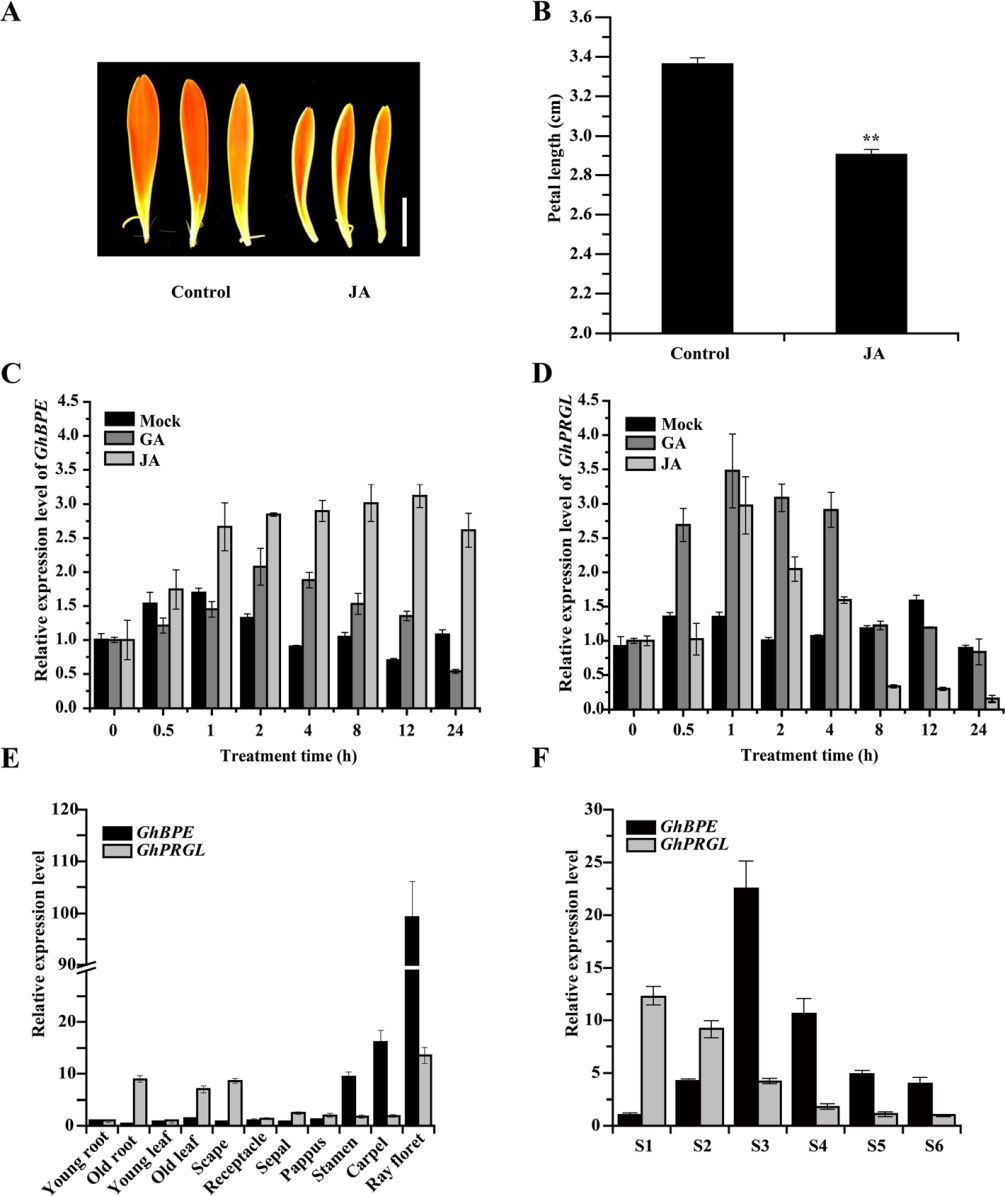
The GhBPE-GhPRGL module regulates ray petal elongation in gerbera via crosstalk between JA and GA. **A** Phenotypes of ray petals treated with 50 μM JA and deionized water (control) for 7 days. Scale bar = 1 cm. **B** Ray petal length after treated with 50 μM JA and deionized water (control) for 7 days. The expression levels of *GhBPE* (**C**) and *GhPRGL* (**D**) in S3 ray petals were detected at different time points (0, 0.5, 1, 2, 4, 8, 12, and 24 h) under the deionized water (control), GA (10 μM), and JA (50 μM) treatments. Relative expression levels of *GhBPE* and *GhPRGL* in different tissues (E) and in ray petal growth stages (S1–S6, where ‘S’ denotes ‘stage’) (**F**). Values are the mean ± SD from three biological replicates. Tukey’s HSD: ** P < 0.01

Li et al. (2015) previously reported that, in gerbera, GA promotes the elongation of ray petals by promoting cell elongation. We tested for an effect of JA and found that it significantly inhibited ray petal size (Fig. 7A, B; Fig. S6). Given that *GhPRGL* is a GASA-family gene whose expression is stimulated by GA (Peng et al., 2008, 2010), and that AtBPEp, a homologue of GhBPE, is a key TF of JA signaling involved in the regulation of late-stage petal elongation (Brioudes et al., 2009), we then tested whether a GA or JA treatment could affect the expression of *GhPRGL* or *GhBPE*. For both genes, their expression profile under the GA treatment showed an increase at early time-points, followed by a decrease later on; JA treatment resulted in different profile, however, one where *GhPRGL* expression rapidly increased at 1 h yet later fell sharply to low levels. In contrast those responses, after its initially rapid increase, *GhBPE* expression remained high for the duration of JA treatment (Fig. 7C, D). These results suggested that, while GA affected the expression of *GhPRGL* and *GhBPE* in a similar manner, JA induced a high level of *GhBPE* expression but ultimately downregulated *GhPRGL*.

Analyzing the spatiotemporal expression patterns of *GhBPE* and *GhPRGL* revealed that either gene was expressed at its peak level in ray floret, indicating a critical role for both GhBPE and GhPRGL in jointly regulating the growth and development in this organ (Fig. 7E). Also, the expression analysis of S1–S6 showed that *GhPRGL* expression waned with ray petal growth, while *GhBPE* expression peaked during S3 (Fig. 7F). In sum, we found that ray petal growth in gerbera is co-regulated by *GhBPE* and *GhPRGL*, while GA and JA operated in concert to regulate the expression of both genes.

## Discussion

### The GhBPE-GhPRGL module regulates ray petal size in gerbera flowers

One of the most prominent characteristics of flowers is their petal size; it is determined by the interaction of genotype and environmental influences, and has important implications for speciation and evolution (Powell and Lenhard, 2012; Krizek and Anderson, 2013; Dennis and Peacock, 2019). Although the external environment has a major influence on the growth and final size of flower organs, it can only function within genetically specified limits (Powell and Lenhard, 2012). In other words, the genetic regulation of flower organs by during their growth and development lays the foundation for the range in attainable petal size. At present, many genes involved in regulating cell proliferation and the transition from cell proliferation to cell expansion have been identified in the model plant, Arabidopsis, and a number of these genes regulate petal size throughout the whole period of petal growth and development (Smyth et al., 1990; Disch et al., 2006; Anastasiou et al., 2007; Krizek and Anderson, 2013; Huang and Irish, 2016; Bowman et al., 1989; Thomson and Wellmer, 2019). In the early stages of petal development, the number of petal cells increases due to cell proliferation, and their cell size tends to remain mostly stable. In the late stages, however, petal cell size increases as a result of two processes: cell growth, which involves an increase in the mass of macromolecules such as proteins and nucleic acids in the cytoplasm, and cell expansion, which entails an increase in vacuole volume (Sugimoto-Shirasu and Roberts, 2003). Studies have shown that the key regulatory point determining the final petal size is the time at which cell proliferation stops in the floral organ primordia (Dinneny et al., 2004; Disch et al., 2006). If cell proliferation ceases too early or too late, it will respectively lead to the diminution or enlargement of flower organs within a given range (Powell and Lenhard, 2012; Krizek and Anderson, 2013). For example, compared with a wild type, the *BB* (*BIG BROTHER*) mutant *bb-1* in Arabidopsis has larger petals due to a longer period of cell proliferation (Disch et al., 2006), whereas the *klu* mutant has smaller petals due to the premature cessation of cell proliferation (Anastasiou et al., 2007). After cell proliferation is halted, cell expansion begins and the cell size increases until petals reach their final size (Sugimoto-Shirasu and Roberts, 2003). For instance, in Arabidopsis, *MED25* mainly constrains final petal size by limiting cell expansion (Xu and Li, 2011), while *MED8* has the opposite function (Xu and Li, 2012). In *Petunia hybrida*, the cell wall protein *PhEXP1* increases petal size because of its role in the cell expansion process (Zenoni et al., 2004, 2011). Although many genes associated with cell proliferation and expansion have been identified through genetic studies, the specific molecular regulatory mechanisms that control cell size and petal growth and development remain largely unknown, especially during cell expansion. In this study, we demonstrated the effects of *GhPRGL*, a gene involved in determining cell size, and its upstream regulator GhBPE, upon the ray petal size of gerbera. In so doing, we uncovered a theoretical basis for the regulatory role of genes in petal growth and development, which should facilitate a better understanding of the molecular and genetic mechanisms underpinning petal growth and development in ornamental flowers.

Because the ray petal is a key component of the flower organ, its associated traits, such as petal size, determine the ornamental features of gerbera. To date, two *GASA* family genes, namely *GEG* and *GhPRGL*, which affect the size of ray petals, have been isolated from gerbera and these two exhibit complementary expression profiles during S1–S6 of petal growth (Kotilainen et al., 1999; Peng et al., 2008, 2010). We found an opposing of *GhPRGL* vis-à-vis *GEG* that can promote ray petal elongation (Fig. 1). Yet petal elongation is finite during petal growth and development, indicating that the role of *GhPRGL* in promoting ray petal elongation must be limited, as indicated by the declining expression of *GhPRGL* during S1–S6 of petal growth, with a particularly low expression level evident at S4–S6 (Fig. 7F). Moreover, by screening a cDNA library of gerbera, we found an upstream TF GhBPE that regulates *GhPRGL* and belongs to the JA signaling pathway; this TF has a very conserved bHLH domain at its N-terminus, much like AtBPEp and AtBPEub (Egea-Cortines et al., 1999). A series of experiments proved GhBPE can bind to the promoter of *GhPRGL* and transcriptionally repress its expression (Fig. 4). Moreover, GhBPE inhibits ray petal elongation by inhibiting cell elongation (Fig. 6A–D), a finding consistent with previous indications that AtBPEp inhibits petal length by inhibiting cell expansion in Arabidopsis (Szécsi et al., 2006). We therefore conclude *GhPRGL* promotes the elongation of ray petals, but this promotion is negatively regulated by GhBPE during ray petal growth; hence, a dynamic equilibrium in the activities of GhPRGL and GhBPE maintains the size of ray petals in gerbera. Previous studies have shown that both GhMIF, a TF that responds to ABA and GA, and GhEIL1, a TF that responds to ethylene, can inhibit ray petal elongation in gerbera by transcriptionally activating *GEG* (Han et al., 2017; Huang et al., 2020). Those authors identified TFs that exert a positive regulatory role in the determination of petal size, while our results revealed a TF having a negative regulatory role, one influenced by phytohormone action. Thus, TFs linked to various hormonal pathways regulate the genes that influence petal size in a coordinated manner.

### Different phytohormones engage in crosstalk to maintain ray petals at a fixed length in gerbera

Because phytohormones are essential for the regulation of cell proliferation and cell expansion (Weiss et al., 2005), they play a role in determining the size of plant organs (Wolters and Jürgens, 2009). For example, cytokinins promote organ growth by stimulating cell proliferation, and their excessive secretion inevitably results in a larger petal size (Bartrina et al., 2011); BR promotes growth by regulating cell division and cell expansion, and so an exogenous application of BR will lead to larger tobacco leaves (Zhang et al., 2021); Auxin, GA, and BR are the key hormones which regulate shade-induced hypocotyl elongation in soybean, while an exogenous application of IAA, GA_3_ and EBL can significantly promote hypocotyl elongation in this plant species (Jiang et al., 2020). Similarly, in Arabidopsis, the elongation of its hypocotyl requires auxin and GA for shade avoidance (Du et al., 2018). In our experiments, ray petal length was significantly reduced after applying the exogenous JA treatment (Fig. 7A, B), indicating that JA has an inhibitory effect on the size of ray petals.

**Fig. 8:**
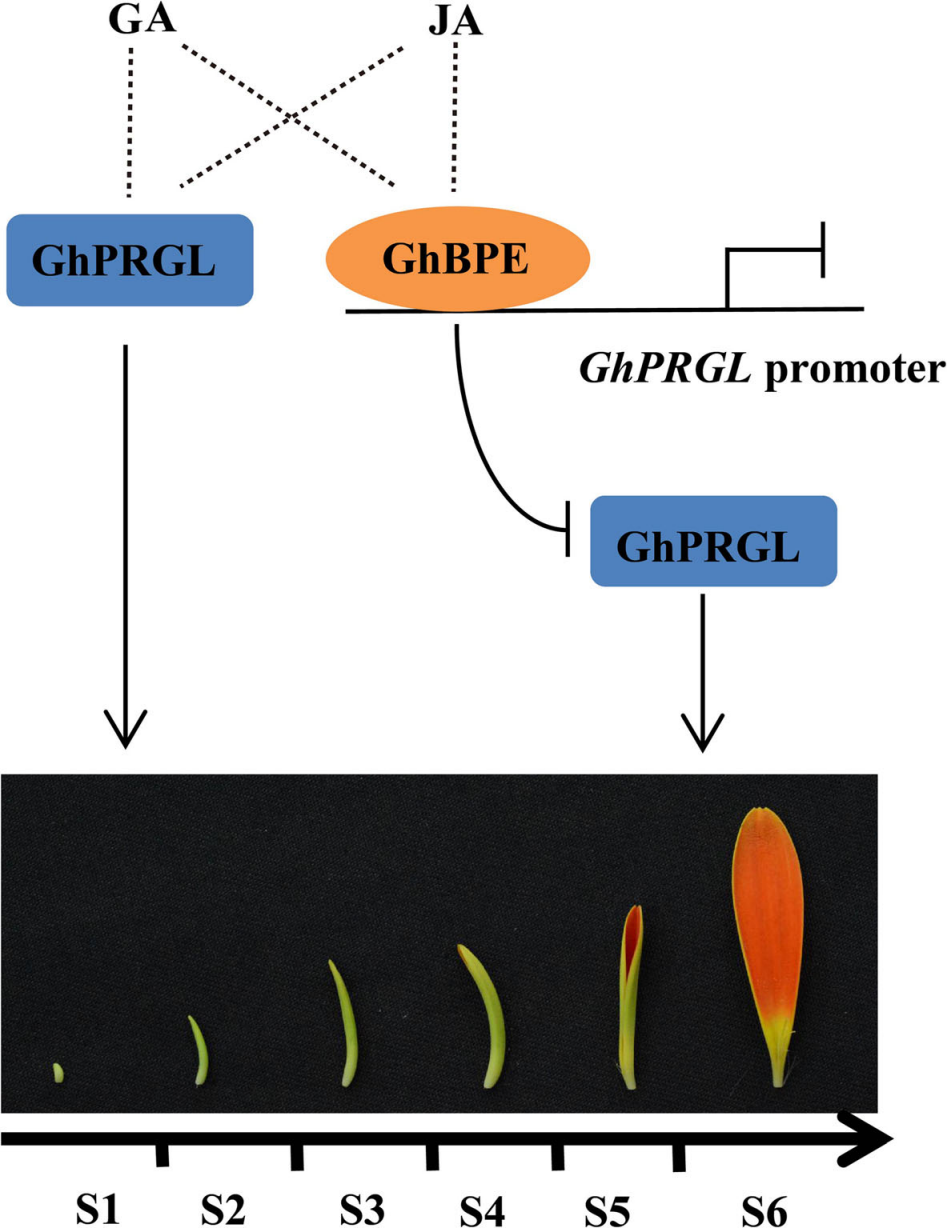
Regulation of the GhBPE-GhPRGL module maintains a fixed ray petal length in gerbera*. GhPRGL* promotes the elongation of ray petals especially in S1–S3, while GhBPE inhibits the elongation of ray petals especially in S3–S6 by transcriptional inhibition of *GhPRGL*. Thus under the coordinated regulation of JA and GA, GhBPE regulates *GhPRGL* to prevent ray petal elongation indefinitely. Solid and dashed lines represent direct and indirect regulation, respectively; the arrows and horizontal lines respectively indicate promotion and inhibition effects

It is well reported that different hormones not only act in isolation, but also act together by engaging in synergistic or antagonistic crosstalk (Wolters and Jürgens, 2009; Jang et al., 2020). The regulatory activity of these hormones can have either positive or negative effects on the growth and development of plants, and this kind of crosstalk among hormone signaling pathways via various regulatory proteins has been extensively studied. For example, numerous TFs in the auxin, ethylene and cytokinin pathways interact to regulate root development in Arabidopsis (Liu et al., 2017); GA and JA antagonistically regulate plant growth and defense through interactions between their key repressors, JAZ and DELLA (Hou et al., 2013; Jang et al., 2020; Liu and Timko, 2021). In a previous study, we found that the elongation of gerbera ray petals is antagonistically regulated by GA and ABA (Li et al., 2015). Crosstalk among GA, ABA, and auxin is mediated by *GhWIP2*, which inhibits cell expansion during petal growth and development in gerbera (Ren et al., 2018). Furthermore, we also discovered that both a GA- and ABA-responsive zinc finger protein and TFs in the ethylene pathway both regulate the size of ray petals via *GASA* family gene (Han et al., 2017; Huang et al., 2020). In the current study, both *GhPRGL* and *GhBPE* responded to GA and JA (Fig. 7C, D). Coupled with the expression patterns of *GhBPE* and *GhPRGL* in petal growth stages (Fig. 7F), we propose a GhBPE-GhPRGL module for the regulation of ray petal size in gerbera. As depicted in Fig. 8, *GhPRGL* promotes the elongation of ray petal especially in S1–S3, while *GhBPE* inhibits the elongation of petals especially in S3–S6 by transcriptional inhibition of *GhPRGL*. This balance of activities, under the coordinated regulation of JA and GA, ensures that ray petals attain a fixed length in each plant.

Mounting studies have shown that the regulation of plant growth and development by JA depends strongly on the crosstalk between JA and other growth-related hormones, such as auxin, cytokinin, GA and BR (de Ollas and Dodd, 2016; Liu et al., 2016; Li et al., 2019; Yang et al., 2019). Our results illustrate how JA and GA can affect the elongation of ray petals in gerbera. In ray petals treated with GA, the expression profile of *GhPRGL* is similar to that of *GhBPE*, although the response of *GhPRGL* is more sensitive. Conversely, these two genes show different expression patterns following the JA treatment (Fig. 7C, D). The *GhPRGL* promoter contains several response elements for certain hormones, such as ABA, BR, ethylene, and auxin (Fig. 2A), which suggest that TFs in other hormone pathways may also activate or repress *GhPRGL*. Thus, additional research exploring the regulation of *GhPRGL* by other TFs in different hormone pathways should provide a more comprehensive picture of how the module that influences final petal size in gerbera is actually regulated.

## Methods

### Plant materials and growth conditions

*Gerbera hybrida* ‘Shenzhen No. 5’ was used in this study. The seedlings were grown under greenhouse conditions: natural light, day and night temperatures respectively of 24°C–28°C and 16°C –20°C, and a relative humidity of 60%–80%. The growth and development of gerbera’s inflorescence consists of six stages (S1–S6) (Meng and Wang, 2004). Ray petals at stages 1.5–3 were used for the transient transformation and hormone treatments.

Arabidopsis seeds (Col-0) were surface sterilized with 75% alcohol and 2.5% sodium hypochlorite, plated on Murashige and Skoog (MS) medium, and then soaked in the dark at 4°C for 3 days for vernalization. Plates were transferred into a greenhouse room (22°C ± 1°C) under long-day conditions (16-h light/8-h dark photoperiod) with a relative humidity of 60%–80% for 7 days. Seedlings were then transferred to a soil medium and grown under the same conditions. The overexpression vector pCanG-GhBPE was generated by cloning the full CDS of *GhBPE* into the vector pCanG. Transgenic Arabidopsis plants were obtained by the floral dip transformation method, as described previously (Su et al., 2016). All primers used in all experiments are listed in Table S1.

### Cloning and bioinformatics analysis of the *GhPRGL* promoter and GhBPE

The promoter sequence of *GhPRGL* was obtained via high-efficiency thermal asymmetric interlaced PCR (Hi-TAIL PCR), as described by Liu and Chen (2007). The promoter sequence was analyzed using the PLACE^1^ and PlantCARE^2^ databases. A full-length *GhBPE* cDNA was amplified from a gerbera cDNA library. Alignment of the deduced amino acid sequences with BPE (BIGPETAL) homologues in different plant species was performed using ClustalX 1.83 and DNAMAN 7.0 software, and their phylogenetic analysis conducted in ClustalX 1.83 and MEGA 6.0. The phylogenetic tree was built using the neighbor-joining algorithm with n = 10,000 bootstrap replicates.

### Subcellular localization

The full CDS of *GhBPE* was cloned into the C17 vector, to generate C17-GhBPE. The C17-GhBPE fusion plasmid and the C17 empty vector were then delivered separately into *N. benthamiana* leaves containing RFP-NLS. The fluorescence signal was detected under a confocal laser-scanning microscope (LSM 710, Zeiss, Germany) after 2 days of culturing.

### Dual-luciferase reporter assay

The dual-luciferase assay was carried out as previously described (Hellens et al., 2005). To characterize the *GhPRGL* core promoter sequence, a series of 5'-deleted fragments were generated, these named *pGhPRGL1732*, *pGhPRGL1339*, *pGhPRGL991*, and *pGhPRGL638* (their numbers represent the base pairs from the end of each fragment to the ATG translation start codon). Then each fragment was fused to pGreenІІ0800 to generate the reporter vector. A modified pBluescript vector (pBS) was used as an effector (Paul et al., 2017). Next, pBS and various reporter plasmids were co-transformed into gerbera protoplasts for the dual-luciferase assay, with pGreenІІ0800 serving as the control.

To analyze whether GhBPE can bind to the *GhPRGL* promoter, the pGreenІІ0800/p*GhPRGL*1732 and pBS/GhBPE vector pairs were co-transformed into gerbera protoplasts, for which the pBS vector and reporter vector were used as controls. To analyze the self-activating activity of GhBPE, *GhBPE* was inserted into an effector vector. The reporter vector contained the firefly *LUC* gene driven by the *CaMV35S* promoter with five tandem copies of *GALRE* upstream; the internal control vector contained the *Renilla LUC* gene driven by the *CaMV35S* promoter. A diagram of the vector constructs is shown in Fig. S3. The plasmids (effectors, reporters, and internal control vectors) were co-transformed into gerbera protoplasts. For each experiment, three biological replicates were used.

### cDNA library construction and yeast one-hybrid (Y1H) Screen

The cDNA library construction was performed by following procedures described by Han et al. (2017). To identify proteins that interact with *pGhPRGL*_*353*_ (at –991 to –638), the cDNA library was introduced into bait strains carrying the pAbAi-p*GhPRGL*_353_ vector. Y1H assays were carried out using the Matchmaker Gold Yeast One-Hybrid Library Screening System (Biosciences Clontech, Palo Alto, CA, USA). GhBPE, a potential interaction protein, was selected for use in further verification. The pGhPRGL_353_-AbAi vector introduced into the Y1H Gold strain was grown on SD-Ura medium to screen for the successful transformation. The prey vector pGADT7-GhBPE was then introduced into the Y1H Gold strain carrying pGhPRGL_353_-AbAi. Transformants were grown on SD/-leu dropout plates containing 200 ng/mL AbA (Aureobasidin A, Clontech, USA), to test for an interaction.

### Yeast two-hybrid (Y2H) system

A Y2H assay was carried out using the Clontech Kit (Matchmaker® Gold Yeast Two-Hybrid System, Cat. No. 630489). The full CDS of *GhBPE* was cloned into the pGBKT7 vector to generate the pGBKT7-GhBPE construct, and this co-transformed into the AH109 yeast strain via an empty pGADT7 vector. Transformed monoclonal yeast cells were first identified on SD-Trp/-Leu medium and then spotted, along with the positive (pGADT7-largeT7/pGBKT7-53) and negative (pGADT7-largeT7/pGBKT7-laminC) controls, onto SD/-Leu/-Trp/-His plates coated with 4 mg/mL X-α-gal. Colonies were incubated at 28°C for 3–4 days in an inverted position and their growth status was noted (Gietz and Schiestl, 2007).

### Electrophoretic mobility shift assay (EMSA)

The EMSA was conducted as described by Han et al. (2017), by using the LightShift Chemiluminescent EMSA kit (Thermo Scientific, USA). The recombinant protein expression vector pET32-GST-GhBPE was grown in *E. coli* BL21 cells and induced overnight with 0.5 mM isopropyl-β-D-thiogalactopyranoside (IPTG) at 25°C. The recombinant protein was purified using a GST fusion protein method with magnetic beads (BBI, China). The 100-bp sequence containing two JA response elements of *pGhPRGL*_*353*_ (–991 to –638) was selected as a 5'-end biotin-labeled probe; the same fragment but unlabeled served as the cold competitor. Mutation probes in which the two JA response elements were mutated with or without labeled biotin constituted the negative controls. Biotin-labeled probes were detected by following the manufacturer’s instructions.

### Transient transformation of ray petals

The transient transformation of ray petals was conducted as described in other recent studies (Han et al., 2017; Huang et al., 2020; Lin et al., 2021). The full CDSs of *GhPRGL* and *GhBPE* were cloned into the pCanG vector to generate the overexpression (OE) vectors pCanG-GhPRGL and pCanG-GhBPE, respectively, while the virus-induced gene silencing (VIGS) vectors pTRV2-GhPRGL and pTRV2-GhBPE were similarly introduced into pTRV2. Every vector (i.e., pCanG, pCanG-GhPRGL, pCanG-GhBPE, pTRV1, pTRV2, pTRV2-GhPRGL, pTRV2-GhBPE) was transformed individually into the *A. tumefaciens* strain C58C1. These *A. tumefaciens* strains were grown on Luria–Bertani (LB) medium containing 75 mg/mL kanamycin and 50 mg/mL rifampicin, for 24 h at 28°C. Next, the cultures were diluted 1:50 (v/v) into 100 mL of LB medium that contained 20 μM acetosyringone (AS), 10 mM 2-(N-morpholinyl) ethanesulfonic acid (MES), 75 mg/mL kanamycin, and 50 mg/mL rifampicin, and then grown overnight at 28°C. When the absorbance (OD600) of *A. tumefaciens* reached 1.5, all cultures were centrifuged at 5,000 g for 5 min and resuspended in an infiltration buffer (10 mM MES, 200 μM AS, 10 mM MgCl_2_, at pH 5.6) to a final OD600 of 1.5. Those *A. tumefaciens* cultures carrying pCanG-GhPRGL and pCanG-GhBPE, and the pCanG vector as the mock, were stored in the dark at room temperature for 4 h. Likewise, *A. tumefaciens* cultures carrying pTRV2-GhPRGL/pTRV2-GhBPE and pTRV1 at a ratio of 1:1 (v/v), and a mixture containing pTRV2/pTRV1 as the mock, were also stored under the same conditions for 4 h.

Ray petals isolated from fresh inflorescences were washed and then immersed in different resuspension buffers under vacuum (–0.09 MPa) for 10 min as described elsewhere (Han et al., 2017; Huang et al., 2020). After 2 min, the vacuum was slowly released, and the petals were washed with sterile distilled water and placed in sterile Petri dishes with two layers of Whatman filter paper. After 3 days of culturing at 4°C, petals were moved into a culture room under long-day conditions (16-h light/8-h dark photoperiod) at 23°C ± 1°C and 50%–60% humidity. Each treatment included at least 30 well-grown petals; three biological replicates were used in each experiment.

### Hormone treatments of ray petals

Ray petals detached from inflorescences at S3 were placed on two layers of Whatman filter papers immersed in JA (50 μM), GA (10 μM), or ddH_2_O. The petals were incubated at 23°C ± 1°C at 50%–60% humidity under long-day conditions (16-h light/8-h dark photoperiod). Sample were collected at 0, 0.5, 1, 2, 4, 8, 12, and 24 h for each treatment and transferred to –80°C for their qRT-PCR analysis. In this experiment, three biological replicates were used.

### Quantitative real-time PCR

Gerbera tissue samples were snap-frozen and transferred to –80°C for their qRT-PCR analysis. Total RNA was extracted from each sample, using the Eastep® Super Total RNA Extraction Kit (Promega, Code No. LS1040), and first-strand cDNA then synthesized according to the manufacturer’s instructions (TOYOBO, Code No. FSQ-301). For quantitative real-time PCR (qRT-PCR), the CFX96 TouchTM Real-Time PCR Detection System (Bio-Rad Laboratories) with the 2× RealStar Green Fast Mixture (GenStar, Code No. A301-01) was used as follows: melting at 95°C for 2 min, then amplification with 40 cycles of 95°C for 5 s and 60°C for 30 s. The data were normalized to the *GhACTIN* (AJ763915) gene, as previously described (Kuang et al., 2013). Three biological replicates and three technical replicates were used. The expression level of a given gene was calculated using the 2^-△△Ct^ method (Livak and Schmittgen, 2001).

### Measurement of ray petal and cell length

Images of gerbera petals were taken using a Nikon D7200 camera (Japan) and their dimensions measured in ImageJ software (http://rsb.info.nih.gov/ij/; NIH, MD, USA). More than 30 petals were selected for length measurements (Li et al., 2015). Their elongation rate was calculated according to Han et al. (2017). To measure the petals’ cell length and number, the top, middle, and basal region of each petal were stained for 30 min with propidium iodide (0.1 mg/mL). Next, images of adaxial epidermal cells were captured under confocal laser scanning microscope (LSM710, Carl Zeiss, Germany) and more than 50 of such cells were analyzed using ImageJ software. At least three biological replicates were used for each observation.

### Statistical analysis

Data were analyzed in SPSS 13.0 software (IBM Corporation, Armonk, NY, USA). Tukey’s honestly significant difference (HSD) test was applied to evaluate statistical significance (* P < 0.05, ** P < 0.01).

### Supplementary Information

**Additional file 1: Fig. S1.** Expression levels of *GhPRGL* in transiently transformed petals at different days. (A) Expression level of *GhPRGL* in *GhPRGL*-OE and the mock at 1–6 days. (B) Expression level of *GhPRGL* in *GhPRGL*-VIGS and the mock at 1–6 days. Each observation was performed with at least three biological replicates. Tukey’s HSD: ** P < 0.01.

**Additional file 2: Fig S2.** GhPRGL promotes the elongation of ray petal cells. Epidermal cell length of *GhPRGL*-OE (A) and *GhPRGL*-VIGS (D) ray petals in the top, middle, and basal region. Epidermal cell width of *GhPRGL*-OE (B) and *GhPRGL*-VIGS (E) ray petals in the top, middle, and basal region. Cell number of epidermal cells per unit area (1 mm^2^) of *GhPRGL*-OE (C) and *GhPRGL*-VIGS (F) ray petals in the top, middle, and basal region. Values are the mean ± SD of three biological replicates. Tukey’s HSD: ** *P* < 0.01.

**Additional file 3: Fig S3.** Diagram of the constructs used for analyzing the transcriptional activity of GhBPE.

**Additional file 4: Fig S4.** Expression levels of *GhBPE* in transiently transformed petals at different days. (A) Expression level of *GhBPE* in *GhBPE*-OE and the mock at 1–9 days. (B) Expression levels of *GhBPE* in *GhBPE*-VIGS and the mock at 1–9 days. Each observation was performed with at least three biological replicates. Tukey’s HSD: ** P < 0.01.

**Additional file 5: Fig. S5.** Phenotypes of *GhBPE*-OE lines in *Arabidopsis*. Inflorescence (A), flowers (B), and petals (C) of 4-week-old *GhBPE*-OE transgenic lines. (D) Petal epidermal cell phenotypes of Col and *GhBPE*-OE lines. #20-5 and #41-5 indicate two independent homozygous lines. Scale bars are 1 cm (A–C) or 50 μm (D).

**Additional file 6: Fig. S6.** Concentration gradient experiment testing JA’s inhibition of ray petal elongation. Ray petal length under different JA concentrations (10 μM, 50 μM, 100 μM, 200 μM, and 500 μM) and deionized water (control) for 7 days. Tukey’s HSD: * P < 0.05.

**Additional file 7: Table S1.** Primers, nucleic acid and amino acid sequences used in this study.

## Data Availability

All data generated or analyzed during this study are included in this published article.

## Abbreviations

ABA: abscisic acid
ABRE: ABA-responsive element
AbA: Aureobasidin A
AS: Acetosyringone
CDS: Coding sequence
Col: Columbia
EBL: 2,4-epibrassinolide
EMSA: Electrophoresis Mobility Shift Assay
GA: gibberellic acid
GARE: GA-responsive element
IAA: indol-3-acetic acid
IPTG: isopropyl-β-D-thiogalactopyranoside
JA: jasmonic acid
LB: Luria-Bertani
MS: Murashige and Skoog
OE: Overexpression
ORF: Open Reading Frame
TF: transcription factor
VIGS: Virus-induced gene silencing
Y1H: Yeast one-hybrid
