# Supplementary figures and images for: A regulatory GhBPE-GhPRGL module maintains ray petal length in Gerbera hybrida
Source: Mol Hortic. 2022 Apr 8;2:9. doi: 10.1186/s43897-022-00030-3 (PMC10515009; doi:10.1186/s43897-022-00030-3)

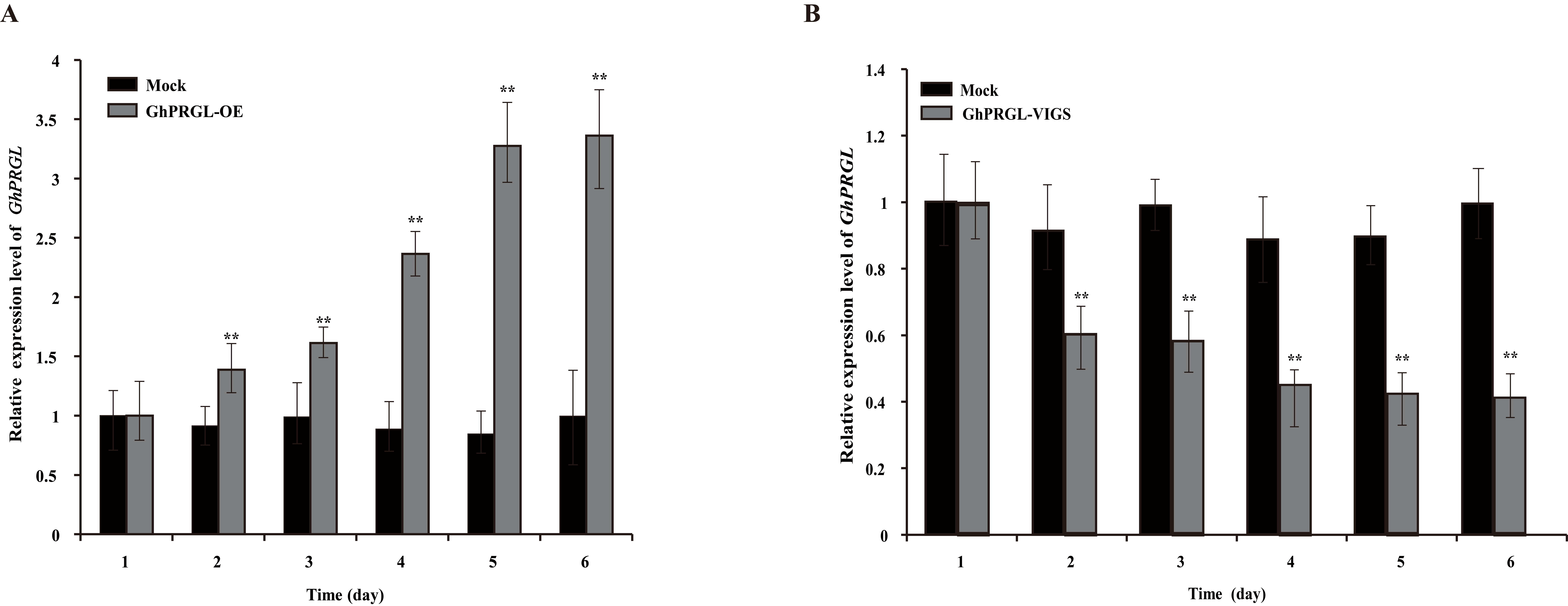

Supplement: Supplementary file 1 — Additional file 1: Fig. S1. Expression levels of GhPRGL in transiently transformed petals at different days. (A) Expression level of GhPRGL in GhPRGL-OE and the mock at 1–6 days. (B) Expression level of GhPRGL in GhPRGL-VIGS and the mock at 1–6 days. Each observation was performed with at least three biological replicates. Tukey’s HSD: ** P < 0.01. [file 43897_2022_30_MOESM1_ESM.jpg]

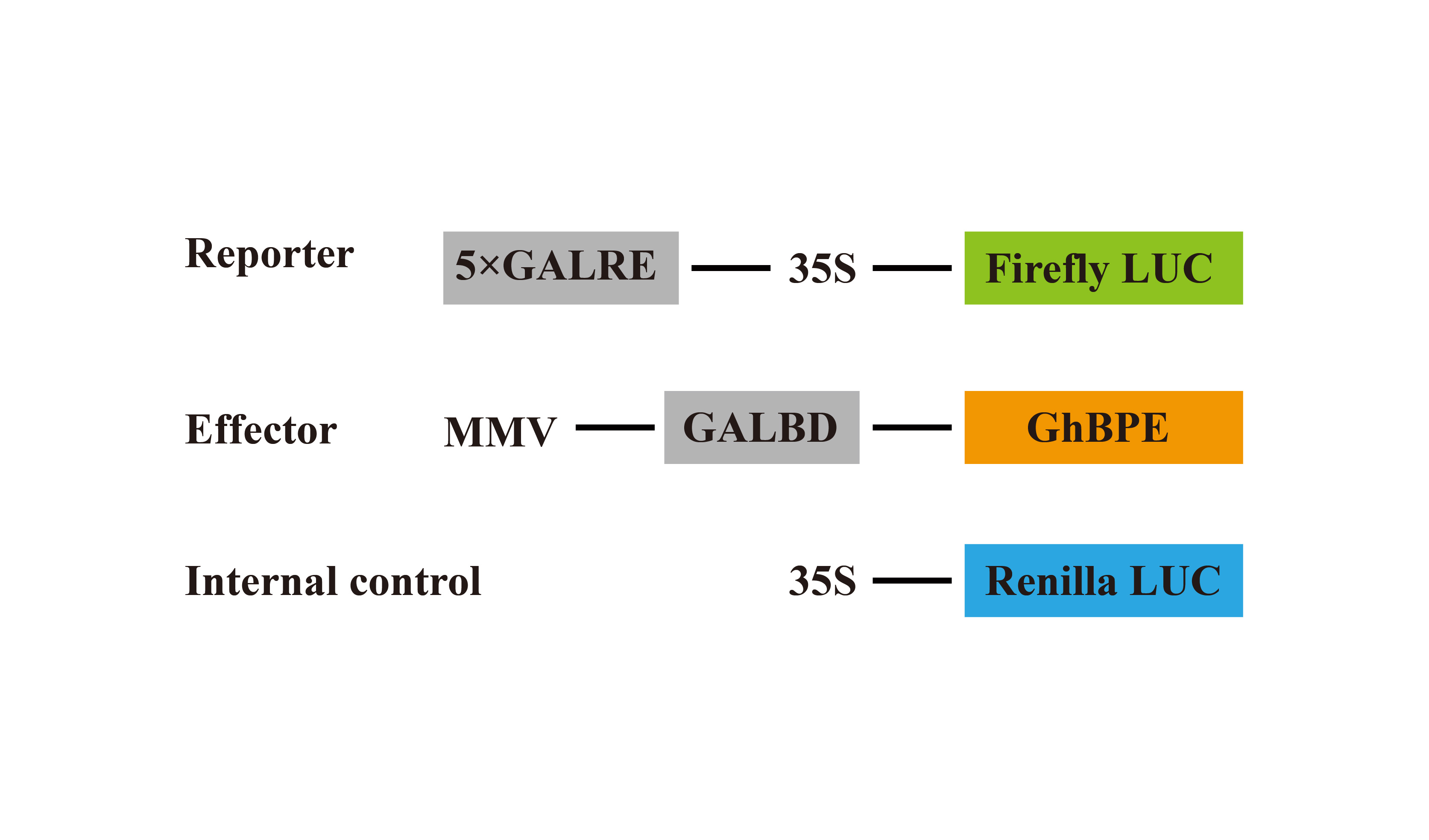

Supplement: Supplementary file 3 — Additional file 3: Fig S3. Diagram of the constructs used for analyzing the transcriptional activity of GhBPE. [file 43897_2022_30_MOESM3_ESM.jpg]

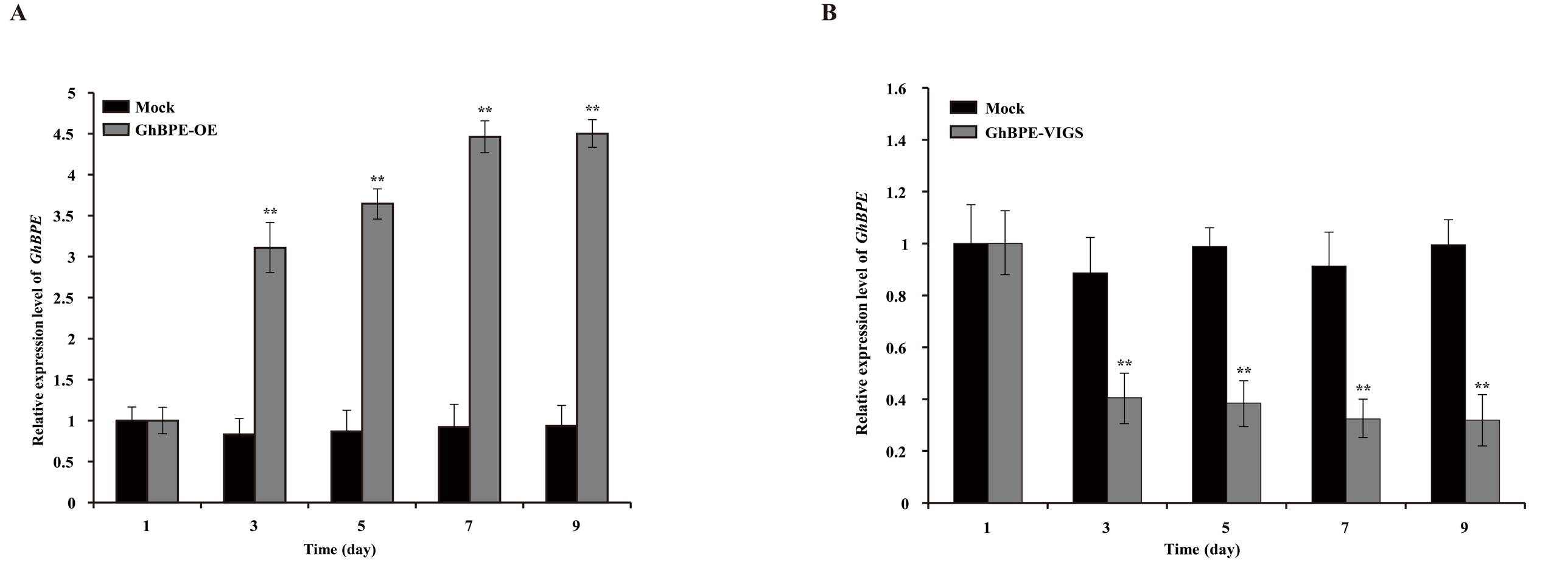

Supplement: Supplementary file 4 — Additional file 4: Fig S4. Expression levels of GhBPE in transiently transformed petals at different days. (A) Expression level of GhBPE in GhBPE-OE and the mock at 1–9 days. (B) Expression levels of GhBPE in GhBPE-VIGS and the mock at 1–9 days. Each observation was performed with at least three biological replicates. Tukey’s HSD: ** P < 0.01. [file 43897_2022_30_MOESM4_ESM.jpg]

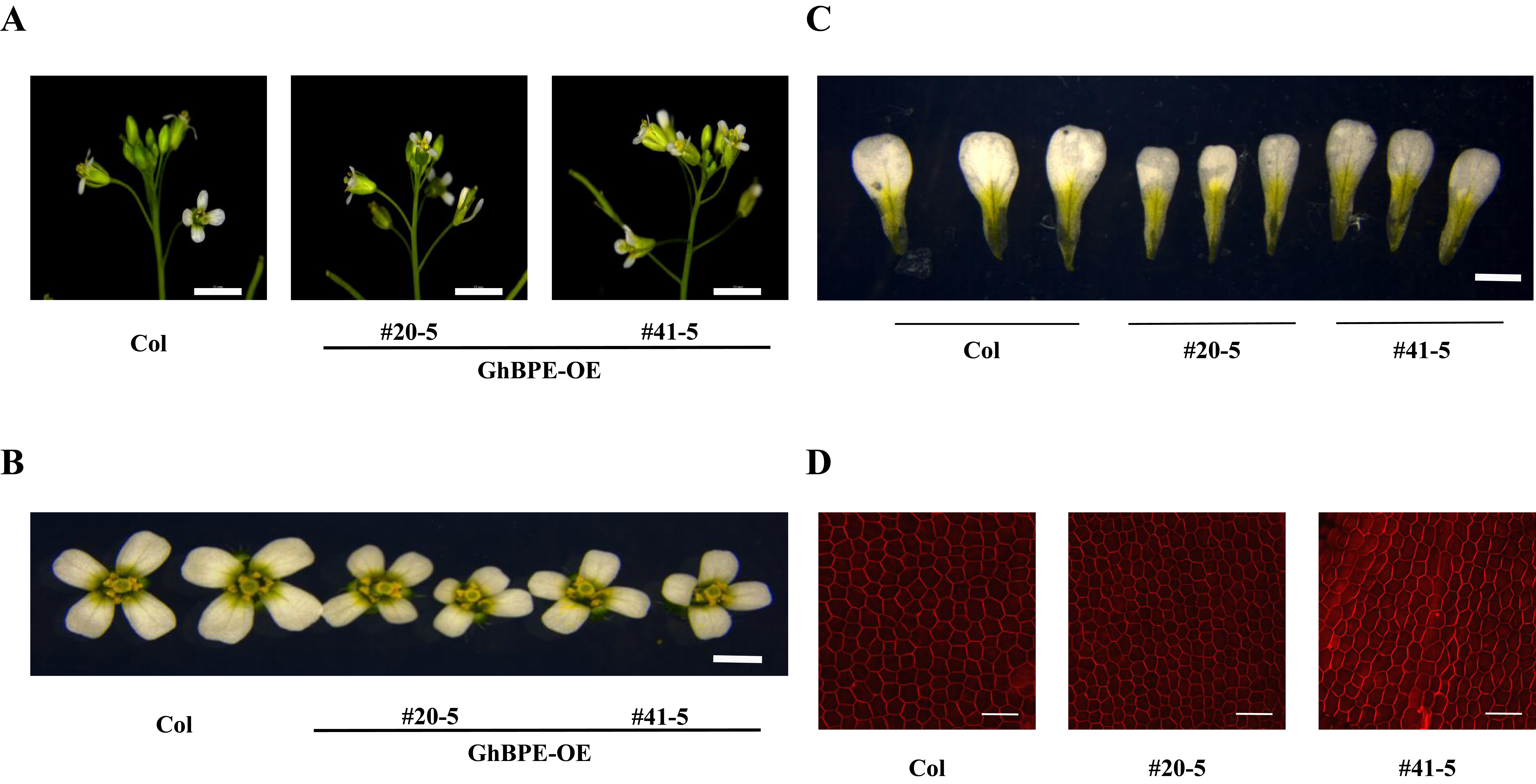

Supplement: Supplementary file 5 — Additional file 5: Fig. S5. Phenotypes of GhBPE-OE lines in Arabidopsis. Inflorescence (A), flowers (B), and petals (C) of 4-week-old GhBPE-OE transgenic lines. (D) Petal epidermal cell phenotypes of Col and GhBPE-OE lines. #20-5 and #41-5 indicate two independent homozygous lines. Scale bars are 1 cm (A–C) or 50 μm (D). [file 43897_2022_30_MOESM5_ESM.jpg]

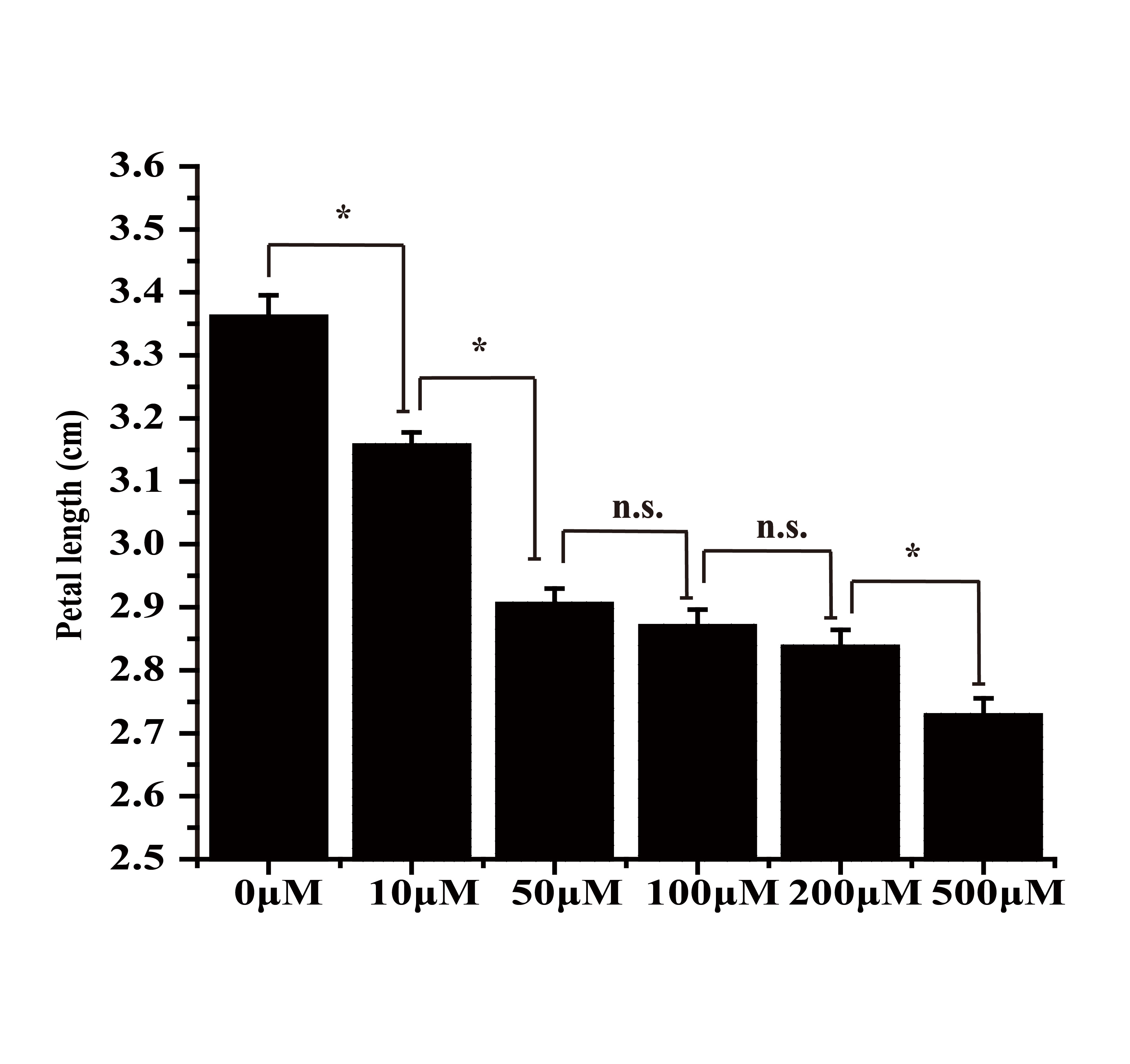

Supplement: Supplementary file 6 — Additional file 6: Fig. S6. Concentration gradient experiment testing JA’s inhibition of ray petal elongation. Ray petal length under different JA concentrations (10 μM, 50 μM, 100 μM, 200 μM, and 500 μM) and deionized water (control) for 7 days. Tukey’s HSD: * P < 0.05. [file 43897_2022_30_MOESM6_ESM.jpg]
